# RecGOBD: accurate recognition of gene ontology related brain development protein functions through multi-feature fusion and attention mechanisms

**DOI:** 10.1093/bioadv/vbae163

**Published:** 2024-11-04

**Authors:** Zhiliang Xia, Shiqiang Ma, Jiawei Li, Yan Guo, Limin Jiang, Jijun Tang

**Affiliations:** Shenzhen Institute of Advanced Technology, Chinese Academy of Sciences, Shenzhen, Guangdong 518055, China; University of Chinese Academy of Sciences, Beijing 100049, China; Shenzhen Institute of Advanced Technology, Chinese Academy of Sciences, Shenzhen, Guangdong 518055, China; Shenzhen Institute of Advanced Technology, Chinese Academy of Sciences, Shenzhen, Guangdong 518055, China; Department of Public Health Sciences, University of Miami, Miami, FL 33136, United States; Department of Public Health Sciences, University of Miami, Miami, FL 33136, United States; Shenzhen Institute of Advanced Technology, Chinese Academy of Sciences, Shenzhen, Guangdong 518055, China

## Abstract

**Motivation:**

Protein function prediction is crucial in bioinformatics, driven by the growth of protein sequence data from high-throughput technologies. Traditional methods are costly and slow, underscoring the need for computational solutions. While deep learning offers powerful tools, many models lack optimization for brain development datasets, critical for neurodevelopmental disorder research. To address this, we developed RecGOBD (Recognition of Gene Ontology-related Brain Development protein function), a model tailored to predict protein functions essential to brain development.

**Result:**

RecGOBD targets 10 key gene ontology (GO) terms for brain development, embedding protein sequences associated with these terms. Leveraging advanced pre-trained models, it captures both sequence and structure data, aligning them with GO terms through attention mechanisms. The category attention layer enhances prediction accuracy. RecGOBD surpassed five benchmark models in AUROC, AUPR, and Fmax metrics and was further used to predict autism-related protein functions and assess mutation impacts on GO terms. These findings highlight RecGOBD’s potential in advancing protein function prediction for neurodevelopmental disorders.

**Availability and implementation:**

All Python codes associated with this study are available at https://github.com/ZL-Xia/RECGOBD.git.

## 1 Introduction

Proteins, as fundamental compounds within living organisms, serve as pivotal structural components and fulfill diverse critical roles in life processes. In the realms of life sciences and medicine, the identification and comprehension of protein functions (Jiang *et al.* 2016) assume paramount significance (Shen *et al.* 2019). The advent of next-generation sequencing technologies has ushered in the ability to rapidly and comprehensively acquire genetic information, including protein sequences (Rives *et al.* 2021), thereby enabling the exploration of unprecedented genetic diversity. However, this technological progress has concurrently posed substantial challenges, particularly in the effective analysis and comprehension of the vast expanse of protein sequence data. As of 6 December 2023, the UniProt (U Consortium 2015, The UniProt Consortium 2021) database houses 570 000 well-annotated protein sequences, underscoring the pressing need for innovative methods to expedite the identification and annotation processes for the 250 million unannotated proteins. In this pursuit, the gene ontology (GO) (G O Consortium 2004, Shen *et al.* 2020) emerges as an invaluable resource. GO, a large-scale bioinformatics database (Huntley *et al.* 2015), categorizes functional terms into three classes (Ashburner *et al.* 2000): molecular function (MF), cellular component (CC), and biological process (BP). The primary objective of protein function prediction lies in assigning proteins to specific GO (Makrodimitris *et al.* 2019, Boadu *et al.* 2023).

Protein function prediction is a complex task that comes with multiple challenges (Clark and Radivojac 2011, Radivojac *et al.* 2013, Yang *et al.* 2015). Firstly, proteins themselves exhibit a high degree of complexity, comprising their amino acid sequences, 3D structures, and multiple functions. The interactions and dependencies between these features add to the difficulty of prediction. Secondly, many proteins do not possess just one function but serve various biological roles, making protein function prediction a multi-label, multi-class problem (Baker and Korhonen 2017, You *et al.* 2018). Addressing this issue requires the ability to recognize and differentiate the multiple functions of proteins while considering their roles in different biological processes and structures. Additionally, protein function can be influenced by factors such as cellular localization, interaction partners (Cao and Cheng 2016, Ding *et al.* 2016), and expression patterns under different conditions. Therefore, accurate function prediction necessitates the integration of various sources of information, including but not limited to sequence data (Hamp *et al.* 2013), structural features, and biological context (Whisstock and Lesk 2003). Therefore, the judicious application of advanced deep learning and machine learning methodologies for predicting protein functions not only offers insights into potential protein functionalities but also greatly facilitates subsequent wet laboratory experiments geared towards validating these predicted functions.

In the field of protein function prediction based on advanced deep learning and machine learning techniques, numerous innovative methods have emerged over the past decade. For example, the naïve (Kulmanov and Hoehndorf 2020) method achieves comparable prediction results by assigning the same GO category to all proteins based solely on annotation frequency. The GO score for a given protein is determined solely by the frequency of its associated GO terms. Following this, methods based on sequence alignment and protein domains, such as BLAST-KNN and LR-Interpro (You *et al.* 2021), are employed. The BLAST-KNN approach operates on the premise that similar proteins share similar functions. Additionally, the LR-Interpro method first uses Interpro (Mitchell *et al.* 2019) to obtain the protein sequence domains, converts them into binary vectors, and then uses logistic regression for training to determine the relationship between proteins and GO terms, thereby predicting protein functions.

For deep learning methods, DeepGO Plus and DeepGraphGO (You *et al.* 2021) are significant approaches. DeepGO Plus (Kulmanov and Hoehndorf 2020) encodes protein sequences directly into 21D one-hot vectors and employs multiple convolutional kernel lengths to extract features. The model concludes with a flat classification layer, resulting in enhanced performance. DeepGraphGO uses domains obtained from Interpro as input and combines the protein–protein interaction network to construct a graph convolutional layer (GCN layer) (Kipf and Welling 2016), capturing higher-order information between nodes (proteins) to predict protein functions.

These innovative methods represent the forefront of protein function prediction, harnessing the capabilities of deep learning and machine learning to decode the multifaceted roles of proteins in biological systems. However, these methods have not yielded particularly strong results in predicting proteins related to brain development. To address this, we propose a novel approach that combines multiple features and employs attention mechanisms to predict protein function specifically related to brain development (Stiles and Jernigan 2010).

In the scope of this investigation, we have devised an innovative protein function prediction methodology designated as RecGOBD (Recognition of Gene Ontology-related Brain Development protein function). This approach seamlessly integrates diverse advanced protein language models (Meier *et al.* 2021) and deploys sophisticated deep learning techniques (Boadu *et al.* 2023, Zhao *et al.* 2024). The inception of our methodology involves the judicious utilization of multiple pre-trained protein language models, encompassing ESM2, ProtBert, Protein2Vec, and one-hot encoding. Our belief lies in the efficacy of integrating structural information from the ESM2 model, contextual information from BERT, local features extracted by Protein2Vec, and positional information from one-hot encoding. This comprehensive fusion approach, utilizing feature-level fusion (Fan *et al.* 2019, Ray *et al.* 2019, Nie *et al.* 2021), entails the direct concatenation of embeddings derived from the protein language models. Following this fusion, a Bi-directional Long Short-Term Memory (Bi-LSTM) network (Zhou *et al.* 2016) is employed to process embedded vectors, enabling the model to glean information bidirectionally within the sequence and enhancing its understanding capabilities. Subsequently, we introduce an attention mechanism meticulously designed to amalgamate protein sequence embeddings with GO labels. This method, harmonizing sequence information with functional annotations (Ofer *et al.* 2021), furnishes a robust tool for more precise and profound protein function prediction. This research constitutes a significant stride in the realm of protein function prediction. By adeptly harnessing multiple models and techniques, it introduces a comprehensive and precise approach, providing a nuanced understanding of the intricacies inherent in protein functionality.

In the culminating phase of our research, we systematically applied our meticulously trained model to predict the impact of mutations at chromosomal gene loci on protein functionality. Our specific focus centered on protein sequence data pertinent to autism spectrum disorder, allowing for a more profound exploration of the intricate relationship between the etiology of autism spectrum disorder and protein function. Initiating this phase, we meticulously gathered protein sequence data directly associated with autism spectrum disorder, encompassing both known proteins linked to the disorder and genes relevant to its pathogenesis. Subsequently, our well-trained model was adeptly employed to forecast the functions of these identified protein sequences. By systematically comparing functional predictions before and after the introduction of mutations, we discerned potential alterations in protein functionality attributed to these genetic variations. This research methodology proves instrumental in uncovering potential causative factors contributing to autism spectrum disorder, particularly by elucidating the nuanced ways in which mutations at chromosomal gene loci impact protein functionality.

## 2 Methods

**Figure 1.**
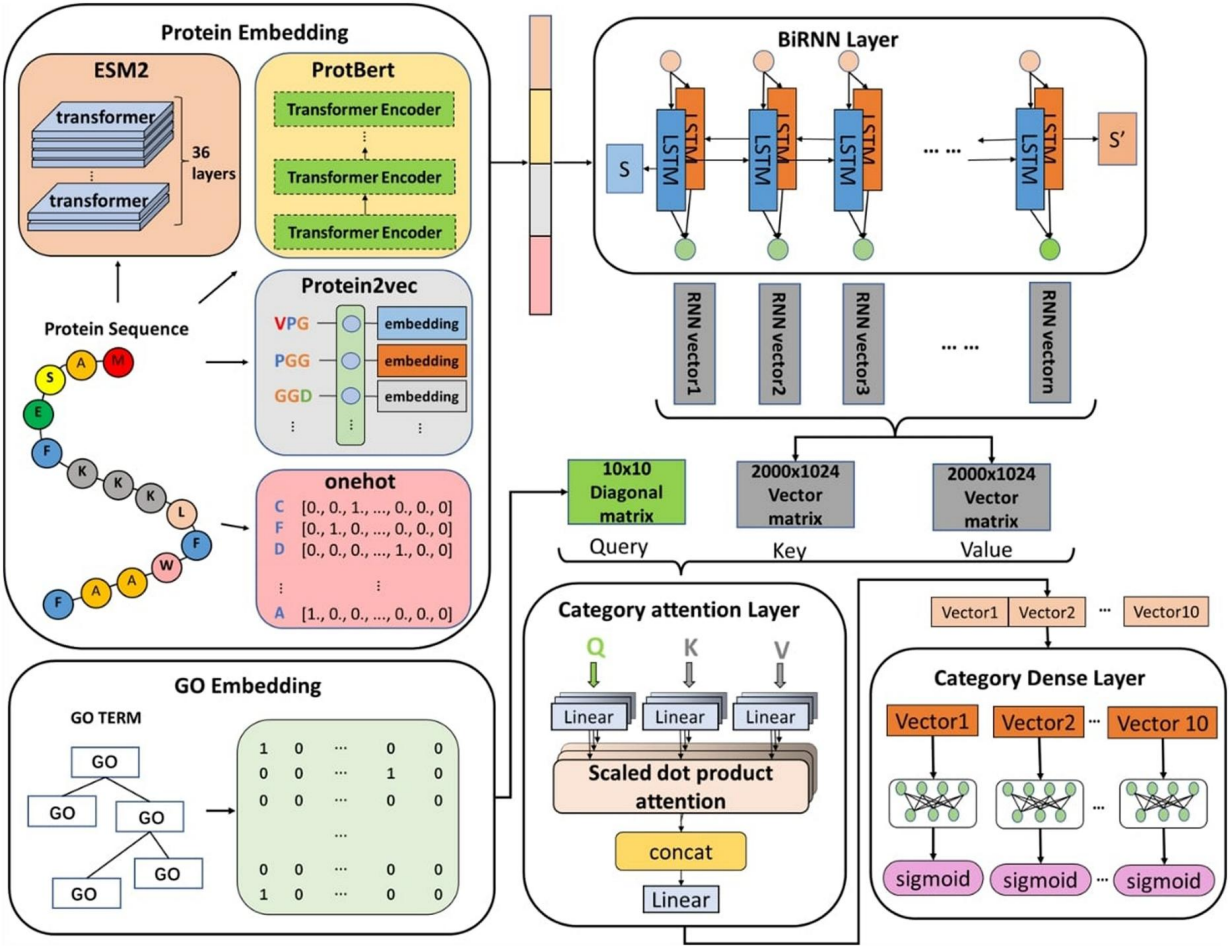
The RecGOBD framework: a four-layer architecture. The first layer of the model is the protein sequence embedding layer, which transforms the original amino acid sequences into dense vector representations, enabling effective processing by the deep learning network. The Bi-LSTM layer captures the deep features between sequences by considering the context of each amino acid in the sequence. The category attention mechanism layer, inspired by attention mechanisms from the field of natural language processing, primarily functions to identify and emphasize the most crucial associations between protein sequences and specific functional labels. The category dense layer then classifies the features extracted and processed by the preceding layers, mapping the complex features learned by the deep network to specific protein function labels, thus providing the final functional prediction for each sequence.

In the course of our research, we have developed an advanced deep learning model specifically designed for predicting protein functions, named RecGOBD (Fig. 1). This model adeptly integrates trained protein language models to extract profound features and discern complex patterns embedded within protein sequences. At its core, the architecture incorporates a Bi-LSTM network, strategically deployed for the effective capture of long-term dependencies inherent in sequence data.

To further augment the model’s discriminative capabilities, we have implemented a category attention mechanism, intensifying the model’s focus on crucial information. Additionally, a category dense layer has been incorporated, facilitating the meticulous processing and classification of potential protein functions. This meticulously designed, multi-layered architecture empowers our model with the precision required to accurately predict the roles proteins play in diverse biological processes, accounting for their potential involvement in multiple functions (Li *et al.* 2021).

### 2.1 Dataset

We utilized a carefully curated dataset focused on proteins related to brain development. Initially, ontologies associated with brain development were selected from GO. Subsequently, protein sequences tagged with these ontologies were retrieved from the Uniprot database. A total of 2200 protein sequences were obtained. These sequences were meticulously extracted and allocated into training, validation, and test sets in an 8:1:1 ratio. The selected GO terms in this article are shown in Table 1.

**Table 1.** GO terms and categories.

GO ID	Categories	Terms
GO:0002250	Biological process	Adaptive immune response
GO:0004984	Molecular function	Olfactory receptor activity
GO:0006955	Biological process	Immune response
GO:0007420	Biological process	Brain development
GO:0042742	Biological process	Defense response to bacterium
GO:0045087	Biological process	Innate immune response
GO:0045471	Biological process	Response to ethanol
GO:0071277	Biological process	Cellular response to Calcium ion
GO:0071456	Biological process	Cellular response to hypoxia
GO:0150104	Biological process	Transport across blood-brain barrier

In this study, the functional attributes of each protein are delineated through a binary vector. Herein, every dimension within the vector corresponds to a distinct label in the GO. This method involves the encoding of each protein sequence into a vector $G_{i}\in {\left\{0,1\right\}}^{1\times d}$, where d denotes the overall number of labels—set at 10 within this research, mirroring the total number of functional categories as defined by GO. Each position within the vector is assigned a value of 1 if the corresponding protein sequence exhibits the particular function associated with that label; conversely, it is assigned a value of 0 if the function is not present.

### 2.2 Protein embedding techniques

We systematically processed each protein sequence through four distinct protein language models to derive comprehensive embeddings. Firstly, ESM2, a 36-layer transformer (Vaswani *et al.* 2017) model with a training dataset of 300 million parameters, was employed to generate 2560D embeddings. Secondly, ProtBert, a model built upon BERT architecture, underwent pre-training on an extensive protein sequence corpus, treating each sequence as an individual document without employing next-sentence prediction. Thirdly, Protein2Vec employed a *k*-mer approach (with *k* = 3), breaking down sequences into 100D vectors, resulting in embeddings of dimensions (*L* − *k* + 1, 100) for proteins with *L* amino acids. Lastly, a simple yet effective one-hot encoding technique was applied to represent the 20 common amino acids as binary vectors, transforming a protein with *L* amino acids into a (*L*, 20) matrix.

#### 2.2.1 The ESM2 embedding

The ESM2 model includes a 36-layer transformer and is pre-trained on large-scale protein sequence data (Vaswani *et al.* 2017, Rives *et al.* 2021), with a training parameter count reaching 3 billion. Protein sequences are processed through a tokenizer, converting amino acids into embeddings. These processed embeddings are fed into the transformer to capture contextual information of protein sequences, including chemical properties and amino acid positions. The transformer comprises encoders and decoders, including multi-head attention mechanisms, feed-forward neural networks, and residual connections. The most crucial component is the attention mechanism, which calculates attention using queries and keys of dimension $d_{k}$, and values of dimension $d_{v}$. *Q*, *K*, *V* represent the Query, Key, and Value matrices, respectively. $W_{i}{}^{Q}$, $W_{i}{}^{K}$, $W_{i}{}^{V}$, and $W^{O}$ are learnable weight matrices, and $d_{k}$ is the dimension of the key

(1) $$\begin{array}{c} \text{MultiHead}(Q,K,V)=\text{Concat}({\text{head}}_{1},\ldots ,{\text{head}}_{h})W^{O}\\ \text{where},{\;\;\text{head}}_{i}=\text{Attention}(QW_{i}^{Q},KW_{i}^{K},VW_{i}^{V}) \end{array}$$

ESM2, renowned for its vast training dataset and the ability to deeply comprehend protein structural information, provides us with in-depth sequence and structural insights.

#### 2.2.2 The ProtBert embedding

The ProtBert, based on the BERT architecture, employs bidirectional transformer networks to learn deep semantic relationships within protein sequences, making it particularly adept at capturing the complexity and diversity of protein sequences.

We utilized ProtBERT from the ProtTrans (Elnaggar *et al.* 2022, Brandes *et al.* 2022) model, a deep learning model specifically designed for protein sequence analysis, trained on the extensive UniRef100 database. Compared to the original BERT model, ProtBERT enhances performance in supervised downstream tasks by increasing the number of layers, while efficiently managing inference time and GPU memory usage. Initially, ProtBERT underwent 300 000 training steps on sequences up to 512 in length, aiding in capturing essential biological features from shorter sequences, like amino acid patterns and local structural information. Subsequently, it was further trained for 100 000 steps on sequences up to 2000 in length, enhancing its ability to process and understand longer sequences. The key feature of the ProtBERT model lies in its generation of deep feature representations, that is, the embeddings of protein sequences, produced through the model’s self-attention mechanism and multi-layered network structure, providing rich contextual information for each amino acid.

#### 2.2.3 The Protein2Vec embedding

In our study, we also employed the Protein2Vec method to encode protein sequences, which is akin to how the Word2Vec model converts words into vector representations (Rong 2014, Zhang *et al.* 2022). The fundamental idea is to map protein sequences into vector representations in a continuous vector space, thereby capturing the semantic similarity and correlation between proteins. These vector representations can be trained using neural network models to transform protein sequences into fixed-dimensional vector representations while preserving sequence information. The Protein2Vec model utilizes this concept by embedding each *k*-mer (a subsequence of length *k*) into a 100D vector space, providing a dense representation for protein sequences. This embedding method captures both the similarity and diversity between *k*-mers, offering rich information for sequence analysis. And we chose 3-mers of specific lengths to encode protein sequences, resulting in each protein sequence being transformed into a fixed-length vector. This encoding method not only helps capture local patterns and features of protein sequences but also facilitates their handling by the deep learning models we constructed.

#### 2.2.4 The one-hot embedding

The one-hot encoding, as a traditional sequence representation method, provides a simple yet direct numerical baseline. In this study, we also employed the one-hot encoding to process protein sequences. This method is a standard technique for converting sequence data into a format easily processed by computational models. In one-hot encoding, each of the 20 common amino acids in the protein sequence is represented as a 20-element vector, where one element is set to 1 and the rest to 0. For example, the amino acid A is encoded as (1, 0, …, 0), F as (0, 1, 0, …, 0), and C as (0, 0, 1, 0, …, 0), etc., giving each amino acid a unique binary representation. When applied to the entire protein sequence, this method can transform a sequence of 2000 amino acids into a 2000 × 20 2D matrix. In this matrix, each row corresponds to a position in the sequence, and each column represents a specific amino acid, with the amino acid at a particular position marked as 1 in its corresponding column. This not only makes the original sequence data more accessible for computational analysis but also preserves the sequential information of the amino acids.

Post-processing, we standardized the sequence lengths to *L*, yielding embeddings of dimensions (*L*, 2560) for ESM2, (*L*, 1024) for ProtBert, (*L*, 100) for Protein2Vec, and (*L*, 20) for one-hot. Subsequently, concatenating these embeddings resulted in a multi-feature fusion embedding of dimensions (*L*, 3704), which demonstrated superior efficacy by complementing distinctive features extracted by the diverse models. Throughout this study, the value of *L* was consistently set to 2000 for standardization and comparative analysis.

### 2.3 Model architecture

After completing the processing of protein sequences and GO labels, we input the embedding of protein sequences, with dimensions (number of sequences, 2000, embedding), into the model. The sequences pass through a Bi-LSTM model (Zhou *et al.* 2016) to obtain contextual information from the combined sequences. This contextual information is transformed into a vector matrix to serve as the Key and Value for the category attention mechanism, while the label matrix is used as the Query. We constructed a 10 × 10 identity matrix, corresponding to 10 GO terms related to brain development. This identity matrix is used as input, multiplied by a weight matrix, and then added to a bias vector, ultimately forming a new query matrix *Q*. The calculation formula is as follows:

(2) $$\begin{array}{c} Q=W^{q}Q_{\text{input}}+b^{q}\\ K=W^{k}K_{\text{input}}+b^{k}\\ V=W^{v}V_{\text{input}}+b^{v}\\ X=Q\cdot K^{⊤}/\sqrt{d_{K}}\\ \hat{X}=\frac{\;\text{exp}\;\left(X_{i,j}\right)}{\sum_{k}\;\text{exp}\;\left(X_{i,k}\right)}\\ B=\hat{X}\cdot V \end{array}$$

where $Q_{\text{input}}$ is the constructed label matrix, and Q is the newly derived query vector. X is the attention score matrix, obtained by the dot product of the query matrix Q and the key matrix K, and then scaled by dividing by $\sqrt{d_{k}}$. $\hat{X}$ is the normalized attention score obtained through the softmax function. B is the output context matrix. For the self-attention layer, Q, K, and V matrices are generated from different inputs. Here, Q is obtained from the label matrix, while K and V are derived from the output of the RNN model processing the protein sequence embeddings.

In this study, we employ a four-head attention mechanism, which outputs 10 vectors each of 400 dimensions. After passing through the category attention layer, we utilize a dense layer to achieve the goal of recognizing protein functions. The output Attention vectors from the attention mechanism are distributed across 10 dense layers, all of which share weights. This approach significantly reduces the number of computational parameters, thereby saving computational resources. The dense layer performs a nonlinear combination of features, with the formula as follows:

(3) Dense(X)=ReLU(WX+b)

where W is the weight matrix and b is the bias vector.

Next, we employ the sigmoid function to calculate the final predicted probabilities for GO, with the formula as follows:

(4) $$\sigma (x)=\frac{1}{1+e^{-x}}$$

where σ(x) is the sigmoid function.

### 2.4 Loss function

We employed two types of loss functions to train our model, comparing them to identify the more effective one. One is the Binary Cross-Entropy loss (BCELoss), and the other is the Focal loss (FocalLoss)

(5) $$\text{BCELoss}=\{\begin{array}{cc} \;-\text{log}\;\left(p(y)\right) & y=1\\ -\text{log}\;\left(1-p(y)\right) & y=0 \end{array}$$

In this context, $y=y_{\text{truth}}\in \{0,1\}\;\text{and}\;p(y)=y_{\text{pred}}\in [0,1].$

(6) $$\text{FocalLoss}=\{\begin{array}{cc} -\alpha \times {\left(1-p(y)\right)}^{\gamma }\times \text{log}(p(y)), & y=1\\ -(1-\alpha )\times p{\left(y\right)}^{\gamma }\times \text{log}(1-p(y)), & y=0 \end{array}$$

In this formula, p(y) represents the model’s predicted probability for the label y, α is a weighting factor to balance positive and negative samples. γ is a focusing parameter that reduces attention to easy-to-classify samples and increases attention to difficult-to-classify samples.

### 2.5 Model training

In our model, we use the PyTorch and TensorFlow frameworks. The pre-trained protein language models are implemented using the PyTorch 1.1.8 framework, while the rest of the models are built using TensorFlow 2.6. Additionally, we train our models using the Nvidia GeForce 3090. Our dataset consists of independent training, validation, and testing sets. We compute sigmoid outputs for 10 labels related to brain development and use the Adam optimizer to minimize the loss function. Our model is fitted on the training set and hyperparameters are optimized on the validation set, with the final performance evaluated on an independent test set. The model employs early stopping, where training is halted if the validation set loss does not decrease within five epochs, ensuring that the experiment does not overfit and retaining the model with the lowest loss for subsequent prediction and evaluation. Each model undergoes experiments with five different random seeds, and the final result of each model is the average of these five trials.

## 3 Results

In this section, we delve into the efficacy of various advanced protein language models in processing protein sequences. Through meticulously designed experiments, we not only evaluate the performance of each model individually but also innovatively fuse the sequences processed by different protein language models to explore their synergistic effects. To comprehensively assess the effectiveness of this fusion strategy, we conducted a series of ablation experiments. By comparing the results under different experimental setups, our aim is to reveal which combination or single model demonstrates optimal performance in processing protein sequences. Finally, we compared our approach with other methods on our brain development-related dataset.

### 3.1 Evaluation metrics

To comprehensively evaluate the performance of our model, we used three evaluation metrics: AUPR (area under the precision-recall curve), AUROC (area under the receiver operating characteristic curve), and Fmax. AUPR is a crucial evaluation metric, particularly for handling imbalanced datasets, as it considers both precision and recall. Precision represents the proportion of true positives among the positive predictions, while recall measures the proportion of true positives predicted among all actual positive cases. On the other hand, AUROC, a widely used performance metric, assesses the model’s diagnostic ability by plotting the relationship between the true positive rate (TPR) and the false positive rate (FPR). The area under the ROC curve (AUROC) provides an effective measure reflecting the overall performance of the model across different decision thresholds. Fmax is the maximum F-measure calculated for all prediction thresholds centered on proteins. First, we use the following formulas to calculate the average precision and recall:

(7) $$\mathrm{pri}(t)=\frac{\sum_{f}I(f\in P_{i}(t)\cap f\in T_{i})}{\sum_{f}I(f\in P_{i}(t))}$$

(8) $$\mathrm{rci}(t)=\frac{\sum_{f}I(f\in P_{i}(t)\cap f\in T_{i})}{\sum_{f}I(f\in T_{i})}$$

(9) $$\text{AvgPr}(t)=\frac{1}{m(t)}\sum_{i=1}^{m(t)}pri(t)$$

(10) $$\text{AvgRc}(t)=\frac{1}{n}\sum_{i=1}^{n}rci(t)$$

where f is the GO term, $T_{i}$ is a set of true annotations, $P_{i}(t)$ is a set of predicted annotations for protein i at threshold t, m(t) is the number of proteins predicted to have at least one class, n is the total number of proteins, and I is an identity function that returns 1 if the condition is true, otherwise 0.

Next, we calculate the maximum $F_{\text{max}}$ value for the prediction threshold:

(11) $$F_{\text{max}}=\underset{t}{\max}\left\{\frac{2\cdot \text{AvgPr}(t)\cdot \text{AvgRc}(t)}{\text{AvgPr}(t)+\text{AvgRc}(t)}\right\}$$

for thresholds t∈[0,1] with a step size of 0.01. We identify the maximum $F_{\text{max}}$ among these thresholds.

### 3.2 Optimizing hyperparameters for enhanced model performance

In our experiments, we specifically focused on three key hyperparameters: learning rate, learning rate scheduler, and loss function, and we thoroughly explored their combined impact on model performance (Table 2). We found that precisely adjusting the learning rate can significantly enhance the model’s performance, especially when processing complex protein sequence data. Additionally, our experimental results revealed a crucial finding: a significant performance improvement can be achieved when four different embedding strategies are fused and applied to the model. Specifically, with the use of the focal loss function along with a learning rate scheduler, the model achieved an AUROC of 0.917, an AUPR of 0.694, and an Fmax of 0.689. On the other hand, when employing the binary cross-entropy (BCE) loss function, the AUROC slightly decreased to 0.914, but the AUPR increased to 0.697 and the Fmax increased to 0.739. These results suggest that after fusing multiple embedding strategies, using a higher learning rate combined with a BCE loss function can yield better outcomes for the model.

**Table 2.** The performance of different embeddings under two types of loss functions.

Model	Loss	Scheduler	Learning rate	AUROC	AUPR	Fmax
one-hot	BCE	None	0.001	0.782	0.427	0.494
	Focal	None	0.001	0.616	0.265	0.409
protein2vec	BCE	None	0.001	0.834	0.51	0.481
	Focal	None	0.001	0.788	0.452	0.473
ProtBert	BCE	None	0.001	0.902	0.614	0.627
	Focal	None	0.001	0.905	0.628	0.564
ESM2	BCE	None	0.001	0.909	0.683	0.702
	Focal	None	0.001	0.908	0.665	0.699
	BCE	None	0.0005	0.905	0.66	0.705
	BCE	StepLR	0.001	0.909	0.677	0.715
ESM2 + one-hot	BCE	None	0.001	0.909	0.67	0.697
	Focal	None	0.001	0.912	0.683	0.701
ESM2 + ProtBert	BCE	None	0.001	0.913	0.68	0.729
	Focal	None	0.001	0.912	0.681	0.726
	BCE	None	0.0005	0.911	0.688	0.635
	BCE	StepLR	0.001	0.915	0.693	0.715
ESM2 + protein2vec + one-hot	BCE	None	0.001	0.913	0.684	0.701
	Focal	None	0.001	0.909	0.673	0.627
ESM2 + ProtBert + one-hot	BCE	None	0.001	0.911	0.667	0.717
	Focal	None	0.001	0.912	0.667	0.712
	Focal	None	0.0005	0.911	0.685	0.660
	Focal	StepLR	0.001	0.913	0.675	0.678
ESM2 + ProtBert + protein2vec + one-hot	BCE	None	0.001	0.914	**0.697**	**0.739**
	Focal	None	0.001	0.915	0.686	0.666
	Focal	None	0.0005	0.913	0.683	0.643
	Focal	StepLR	0.001	**0.917**	0.694	0.689

Note: Bold fonts indicate the best results.

### 3.3 Comparative analysis of single protein sequence embedding technologies

**Figure 2.**
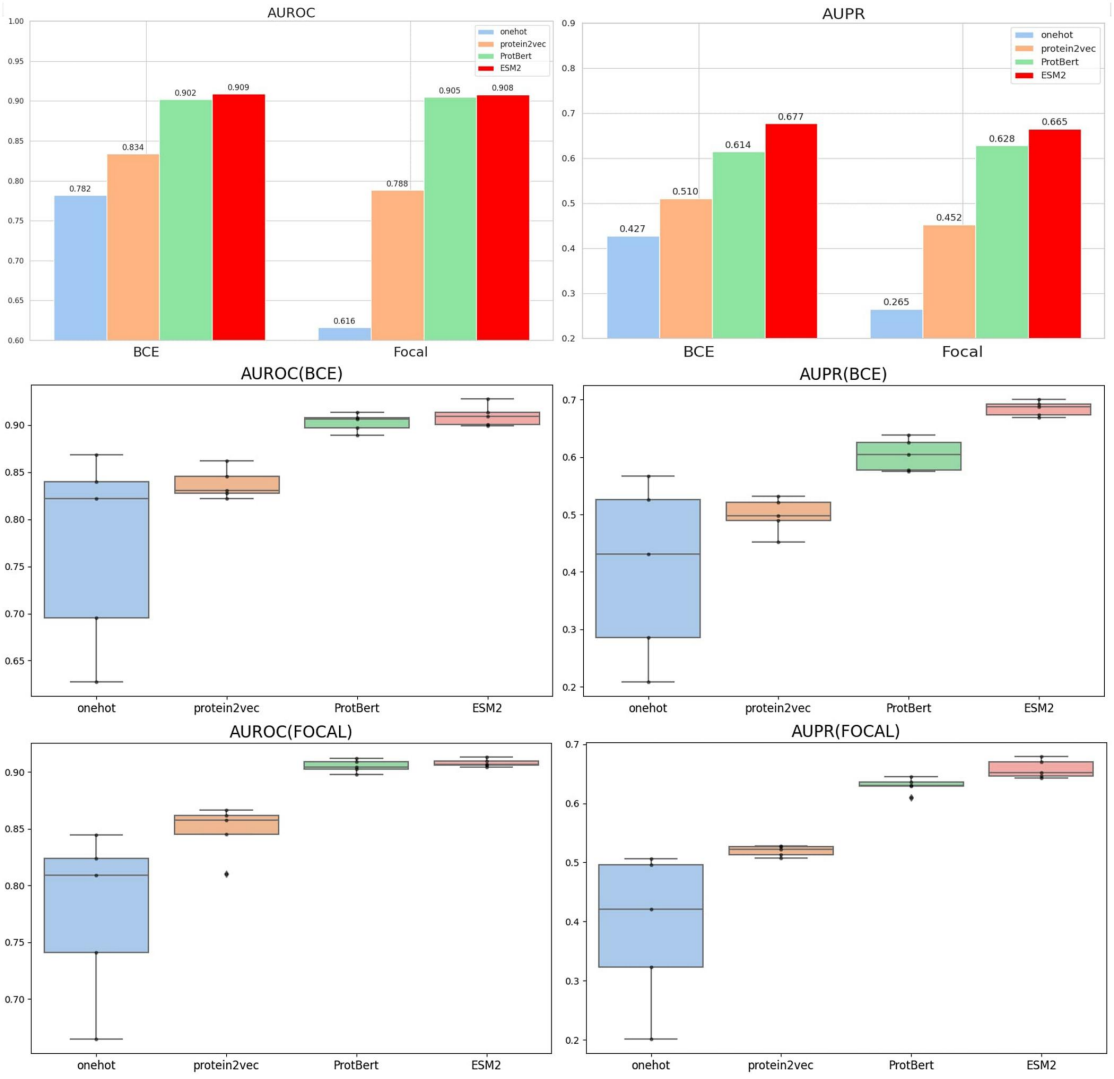
Comparative analysis of single protein sequence embedding technologies. The top two bar charts display the performance of average AUROC and average AUPR under two different loss functions (BCE and FOCAL) under the same conditions. The box plots below illustrate the distribution of specific AUROC and AUPR values for BCE and FOCAL, from which we can conclude that the ESM model achieves the best average AUPR and AUROC performance for individual protein sequence embeddings, and also demonstrates greater stability.

We conducted an in-depth analysis of the performance of four different protein sequence embedding techniques (one-hot encoding, protein2vec, ProtBert, and ESM2) under various fusion configurations. We evaluated the performance of these embedding technologies when used alone and in combination with different loss functions and learning rate schedulers (Fig. 2). Specifically, using one-hot encoding under BCE loss, the model’s AUROC was 0.782, the AUPR was 0.427, and the Fmax was 0.494. With protein2vec, auroc and aupr improved to 0.834 and 0.51, respectively. The application of ProtBert increased the AUROC, AUPR, and Fmax to 0.902, 0.614, and 0.627, respectively, while switching to Focal loss slightly improved these values to 0.905, 0.628, and 0.564. ESM2 showed the strongest individual performance under BCE loss, with an AUROC of 0.909, an AUPR of 0.683, and Fmax of 0.702. When only using a single pre-trained model for protein sequence embedding, it can be concluded that the ESM2 model yields better prediction results because it can extract features inherent to the sequence itself and tighter relationships between sequences.

We conducted a comparative analysis of correlation for various embedding technologies. The detailed results are provided in Section 5 of the Supplementary File.

### 3.4 Comparative analysis of protein sequence embedding combinations

**Figure 3.**
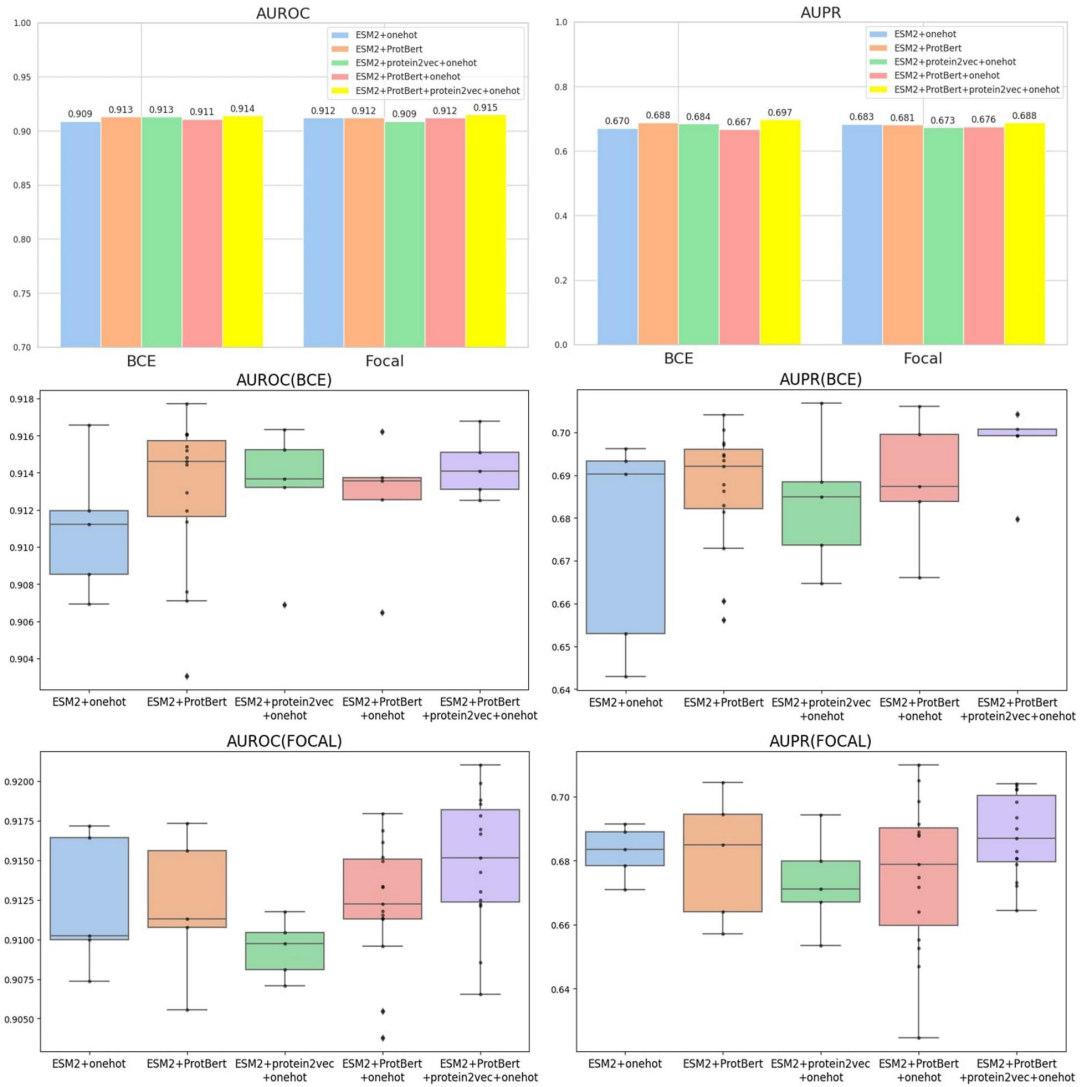
Comparative analysis of protein sequence embedding combinations. The top two bar charts demonstrate the average AUROC and AUPR performance under BCE and FOCAL loss functions after combining multiple embedding techniques. This indicates that compared to using a single embedding method, the combination of multiple embedding approaches significantly enhances predictive capabilities, with the amalgamation of four different embeddings yielding the best outcomes. The box plots below detail the distribution of AUROC and AUPR under BCE and FOCAL conditions, revealing that the model’s predictive performance is superior and the results for AUROC and AUPR are more stable when the four embedding methods are combined.

We conducted a comprehensive performance analysis of combinations comprising four distinct protein sequence embedding techniques: one-hot encoding, protein2vec, ProtBert, and ESM2, across a range of fusion configurations (Fig. 3). The combination of ESM2 and ProtBert achieved the best performance with BCE loss and StepLR scheduler, reaching an AUROC of 0.915, an AUPR of 0.693, and an Fmax of 0.715. Ultimately, when we combined all four embeddings (one-hot, protein2vec, ProtBert, and ESM2) and operated the model under BCE loss and learning rate of 0.001, we observed the best results, with an AUROC of 0.914, an AUPR of 0.697, and an Fmax of 0.739. Although the AUROC was not the highest, the AUPR and Fmax metrics were sufficiently impressive. We conducted a detailed statistical analysis and also utilized graphical representations for intuitive visualization. Particularly, when combining the four embedding techniques (one-hot encoding, protein2vec, ProtBert, and ESM2), we observed that this configuration excelled in performance metrics, achieving optimal metrics value. These results indicate that the model with combined embedding technologies not only achieves high performance but also exhibits remarkable stability and consistency. We also conducted a specific GO performance analysis, the details of which are provided in Section 6 of the Supplementary File.

### 3.5 Comparison with other methods

We carefully selected GO terms closely associated with brain development and extracted 2200 corresponding protein sequences. These sequences were divided into training, validation, and test sets in an 8:1:1 ratio. The model configuration of RecGOBD that performed best on the validation set was selected for testing. This model utilizes a fusion of four features, employs BCEloss as the loss function, and is set with a learning rate of 0.001. To comprehensively evaluate the performance of our proposed RecGOBD model, we compared it against five other methods using AUROC, AUPR, and Fmax metrics. The results are presented in Table 3. Notably, the LR-Interpro method excels in AUROC and AUPR, while the RecGOBD model outperforms all other methods in all evaluation metrics, particularly achieving an Fmax of 0.683. This outcome highlights the significant advantages of our RecGOBD model in predicting protein functions related to brain development.

**Table 3.** Performance comparison on test dataset.

Methods	AUROC	AUPR	Fmax
Naive	0.678	0.189	0.306
BLAST-KNN	0.500	0.117	0.209
LR-Interpro	0.892	0.577	0.594
DeepGOplus	0.613	0.258	0.244
DeepGraphGO	0.500	0.120	0.214
RecGOBD	**0.916**	**0.639**	**0.683**

Note: Bold fonts indicate the best results.

### 3.6 Exemplary applications of RecGOBD in autism spectrum disorders

In our research, we adopted an integrated approach to explore genetic mutations on the human genome and their impact on protein functions. Specifically, we first downloaded extensive data from the 22 autosomes of the human genome and carefully selected and extracted 10 838 amino acid sequences (Turner *et al.* 2017, An *et al.* 2018). These sequences represent the chromosomal state before and after mutations. By analysing each sequence, we accurately determined the precise locations of these mutations. Subsequently, we input these pre- and post-mutation amino acid sequences into our previously trained predictive model, which integrates four different embedding methods. This approach allowed us to compare the functional differences of the same protein before and after mutation, focusing particularly on changes in GO annotations. These annotations provide valuable information about the biological processes, cellular components, and molecular functions the protein may be involved in. Ultimately, we identified 2484 amino acid sequences that underwent GO annotation changes due to mutations. We traced these mutation sites to their corresponding gene names, exploring the association between gene changes and amino acid site mutations, and determining whether gene functions were gained or lost due to these mutations. This not only aids in our deeper understanding of the impact of genetic mutations on organisms but also holds significant implications for future gene therapy and precision medicine research. Our partial predictive results are presented in Table 4. The complete table can be found in Supplementary Table S1.

**Table 4.** Changes in GO caused by chromosomal variations.

Chromosome	Variant	Gene name	GO ID	GO term	Category	Gain/loss
chr1	C1645227CG	CDK11B	GO:0002250	Adaptive immune response	BP	Gain
chr1	T2412474TG, G2406766A	PEX10	GO:0071456	Cellular response to hypoxia	BP	Loss
chr1	C12755727T	CFAP107	GO:0006955	Immune response	BP	Loss
chr2	CA20006084C	MATN3	GO:0071456	Cellular response to hypoxia	BP	Loss
chr2	AG32209510A	SLC30A6	GO:0045471	Response to ethanol	BP	Gain
chr3	C9928346T	IL17RC	GO:0006955	Immune response	BP	Loss
chr3	AC15644492A	BTD	GO:0006955	Immune response	BP	Gain
chr4	TTCTC36284187T	DTHD1	GO:0007420	Brain development	BP	Loss
chr4	C75921108A	NAAA	GO:0071456	Cellular response to hypoxia	BP	Loss
chr5	GAA141388491G	PCDHGA1	GO:0007420	Brain development	BP	Loss
chr5	G140632441GT	CD14	GO:0045471	Response to ethanol	BP	Loss
chr6	CAG30584533C	ABCF1	GO:0045087	Innate immune response	BP	Loss
chr6	GA73466245G	MTO1	GO:0071456	Cellular response to hypoxia	BP	Gain
chr7	G47978506A	HUS1	GO:0006955	Immune response	BP	Gain
chr7	G76054918GC	MDH2	GO:0006955	Immune response	BP	Gain
chr8	CA123183070C	FAM83A	GO:0007420	Brain development	BP	Gain
chr8	C95263752CT	CFAP418-AS1	GO:0002250	Adaptive immune response	BP	Loss
chr9	GCTGAA92324075G	NOL8	GO:0002250	Adaptive immune response	BP	Gain
chr9	T128250832TG	DNM1	GO:0007420	Brain development	BP	Gain
chr10	C26203058T	MYO3A	GO:0002250	Adaptive immune response	BP	Gain
chr10	T43158796TG	CSGALNACT2	GO:0006955	Immune response	BP	Gain
chr10	GA84470419G	CCSER2	GO:0007420	Brain development	BP	Loss
chr11	T614897TGCGCAGC	IRF7	GO:0071456	Cellular response to hypoxia	BP	Gain
chr11	CCT31632359C	ELP4	GO:0007420	Brain development	BP	Gain
chr11	CAT34108759C	NAT10	GO:0002250	Adaptive immune response	BP	Gain
chr12	CT21491612C	RECQL	GO:0006955	Immune response	BP	Loss
chr12	AT32722450A	DNM1L	GO:0045471	Response to ethanol	BP	Loss
chr13	AG96987649A	OXGR1	GO:0006955	Immune response	BP	Loss
chr13	GCTGA108210668G	LIG4	GO:0007420	Brain development	BP	Gain
chr14	AC22773685A	SLC7A7	GO:0150104	Transport across blood–brain barrier	BP	Gain
chr14	CT23097830C	C14orf119	GO:0071456	Cellular response to hypoxia	BP	Gain
chr14	TG75633685T	FLVCR2	GO:0071456	Cellular response to hypoxia	BP	Gain
chr15	GT78923022G	CTSH	GO:0071456	Cellular response to hypoxia	BP	Loss
chr15	AT81334731A	TMC3-AS1	GO:0007420	Brain development	BP	Gain

In the table, the first column, ‘Chromosome’ specifies the particular chromosome within the human genome where the mutation occurs. The second column, ‘Variant’ details the specific mutation at a designated chromosomal position, such as ‘C1645227CG’ which indicates a change from ‘C’ to ‘CG’ at position 1645227. The third column, ‘Gene Name’ identifies the gene linked to the mutation at that locus, for instance, ‘CDK11B’ corresponds to the mutation at position 1645227 on chromosome 1. The fourth and fifth columns, ‘GO ID’ and ‘GO Term’, list the GO identifiers and their descriptions, respectively. The sixth column, ‘Category’ specifies the biological category associated with each GO term. The final column, ‘Gain/Loss’ denotes whether the mutation resulted in a gain or loss of the GO annotation for the protein due to the genetic alteration.

Due to the extensive length of the protein sequences, they are not fully displayed in Table 4. Instead, they are included in Supplementary Table S1, which features two additional columns: ‘Ref’ representing the protein sequence before mutation, and ‘Alt’ showing the protein sequence post-mutation.

## 4 Conclusion

In our research, we have developed RecGOBD, a model founded on attention mechanisms, which integrates multiple pre-trained protein language models to enhance the functional prediction of protein sequences. Initially, protein sequences undergo processing through the pre-trained models utilizing four embedding technologies, resulting in embeddings. These embeddings subsequently traverse a Bi-LSTM network, culminating in an attention mechanism that elucidates the relationship between protein sequences and GO annotations. This attention mechanism, combined with a dense layer for categorization, enables our model to proficiently execute multi-label functional prediction.

To demonstrate RecGOBD’s performance, we compared the model with five existing deep learning and machine learning methods, and RecGOBD outperformed the others across all performance metrics. And then we applied it to autism spectrum disorder disease, commencing with mutation analysis to unveil associations between mutations and protein GO function. By analysing correlations between locus variations and resultant functional changes, our approach facilitates a targeted exploration of protein functional pathways linked to autism spectrum disorder. This contributes valuable insights that advance ongoing research efforts centered on brain disorders.

The true value of our model lies in its capacity to serve as a reference for future experimental endeavors, providing a framework for manual experimentation. Leveraging the insights gleaned from our model, researchers can conduct experiments more effectively and economically, expediting the process of determining accurate protein functions.

## Supplementary Material

vbae163_Supplementary_Data

## References

[vbae163-B2] An J-Y , Lin K, Zhu L et al Genome-wide de novo risk score implicates promoter variation in autism spectrum disorder. Science 2018;362:eaat6576. 30545852 10.1126/science.aat6576 PMC6432922

[vbae163-B3] Ashburner M , Ball CA, Blake JA et al Gene ontology: tool for the unification of biology. Nat Genet 2000;25:25–9. 10802651 10.1038/75556 PMC3037419

[vbae163-B4] Baker S , Korhonen A. 2017. Initializing Neural Networks for Hierarchical Multi-Label Text Classification. Kerrville, TX, USA: Association for Computational Linguistics.

[vbae163-B5] Boadu F , Cao H, Cheng J. Combining protein sequences and structures with transformers and equivariant graph neural networks to predict protein function. Bioinformatics 2023;39(Suppl 1):i318–25. 37387145 10.1093/bioinformatics/btad208 PMC10311302

[vbae163-B6] Brandes N , Ofer D, Peleg Y et al ProteinBERT: a universal deep-learning model of protein sequence and function. Bioinformatics 2022;38:2102–10. 35020807 10.1093/bioinformatics/btac020 PMC9386727

[vbae163-B7] Cao R , Cheng J. Integrated protein function prediction by mining function associations, sequences, and protein–protein and gene–gene interaction networks. Methods 2016;93:84–91. 26370280 10.1016/j.ymeth.2015.09.011 PMC4894840

[vbae163-B8] Clark WT , Radivojac P. Analysis of protein function and its prediction from amino acid sequence. Proteins: Struct, Funct, Bioinform 2011;79:2086–96. 10.1002/prot.23029 21671271

[vbae163-B9] Consortium, Gene Ontology. The gene ontology (GO) database and informatics resource. Nucleic Acids Res 2004;32:D258–61. 14681407 10.1093/nar/gkh036 PMC308770

[vbae163-B10] Consortium, UniProt. UniProt: a hub for protein information. Nucleic Acids Res 2015;43:D204–12. 25348405 10.1093/nar/gku989 PMC4384041

[vbae163-B11] Ding Y , Tang J, Guo F. Predicting protein–protein interactions via multivariate mutual information of protein sequences. BMC Bioinformatics 2016;17:398–13. 27677692 10.1186/s12859-016-1253-9 PMC5039908

[vbae163-B1] Elnaggar A , Heinzinger M, Dallago C et al ProtTrans: Toward Understanding the Language of Life Through Self-Supervised Learning. IEEE Trans Pattern Anal Mach Intell 2022;44:7112–127. 34232869 10.1109/TPAMI.2021.3095381

[vbae163-B12] Fan W , He Z, Xing X et al Multi-modality depression detection via multi-scale temporal dilated CNNS. In: *Proceedings of the 9th International on Audio/Visual Emotion Challenge and Workshop*, Nice, France. New York, NY, United States: Association for Computing Machinery, 2019, 73–80.

[vbae163-B13] Hamp T , Kassner R, Seemayer S et al Homology-based inference sets the bar high for protein function prediction. BMC Bioinformatics 2013;14 Suppl 3:S7–10. 10.1186/1471-2105-14-S3-S7 PMC3584931 23514582

[vbae163-B14] Huntley RP , Sawford T, Mutowo-Meullenet P et al The Goa database: gene ontology annotation updates for 2015. Nucleic Acids Res 2015;43:D1057–63. 25378336 10.1093/nar/gku1113 PMC4383930

[vbae163-B15] Jiang Y , Oron TR, Clark WT et al An expanded evaluation of protein function prediction methods shows an improvement in accuracy. Genome Biol 2016;17:184–19. 27604469 10.1186/s13059-016-1037-6 PMC5015320

[vbae163-B16] Kipf TN , Welling M. Semi-supervised classification with graph convolutional networks. arXiv, arXiv:1609.02907, 2016, preprint: not peer reviewed.

[vbae163-B17] Kulmanov M , Hoehndorf R. DeepGOPlus: improved protein function prediction from sequence. Bioinformatics 2020;36:422–9. 31350877 10.1093/bioinformatics/btz595 PMC9883727

[vbae163-B18] Li J , Pu Y, Tang J et al DeepATT: a hybrid category attention neural network for identifying functional effects of DNA sequences. Brief Bioinform 2021;22:bbaa159. 32778871 10.1093/bib/bbaa159

[vbae163-B19] Makrodimitris S , van Ham RC, Reinders MJT. Improving protein function prediction using protein sequence and GO-term similarities. Bioinformatics 2019;35:1116–24. 30169569 10.1093/bioinformatics/bty751 PMC6449755

[vbae163-B20] Meier J , Rao R, Verkuil R et al Language models enable zero-shot prediction of the effects of mutations on protein function. Adv Neural Inform Process Syst 2021;34:29287–303.

[vbae163-B21] Mitchell AL , Attwood TK, Babbitt PC et al InterPro in 2019: improving coverage, classification and access to protein sequence annotations. Nucleic Acids Res 2019;47:D351–60. 30398656 10.1093/nar/gky1100 PMC6323941

[vbae163-B22] Nie W , Yan Y, Song D et al Multi-modal feature fusion based on multi-layers LSTM for video emotion recognition. Multimed Tools Appl 2021;80:16205–14.

[vbae163-B23] Ofer D , Brandes N, Linial M. The language of proteins: NLP, machine learning & protein sequences. Comput Struct Biotechnol J 2021;19:1750–8. 33897979 10.1016/j.csbj.2021.03.022 PMC8050421

[vbae163-B24] Radivojac P , Clark WT, Oron TR et al A large-scale evaluation of computational protein function prediction. Nat Methods 2013;10:221–7. 23353650 10.1038/nmeth.2340 PMC3584181

[vbae163-B25] Ray A , Kumar S, Reddy R et al Multi-level attention network using text, audio and video for depression prediction. In: *Proceedings of the 9th International on Audio/Visual Emotion Challenge and Workshop*, Nice, France. New York, NY, United States: Association for Computing Machinery, 2019, 81–88.

[vbae163-B26] Rives A , Meier J, Sercu T et al Biological structure and function emerge from scaling unsupervised learning to 250 million protein sequences. Proc Natl Acad Sci USA 2021;118:e2016239118. 33876751 10.1073/pnas.2016239118 PMC8053943

[vbae163-B27] Rong X. word2vec parameter learning explained. arXiv, arXiv:1411.2738, 2014, preprint: not peer reviewed.

[vbae163-B28] Shen Y , Ding Y, Tang J et al Critical evaluation of web-based prediction tools for human protein subcellular localization. Brief Bioinform 2020;21:1628–40. 31697319 10.1093/bib/bbz106

[vbae163-B29] Shen Y , Tang J, Guo F. Identification of protein subcellular localization via integrating evolutionary and physicochemical information into Chou’s general PseAAC. J Theor Biol 2019;462:230–9. 30452958 10.1016/j.jtbi.2018.11.012

[vbae163-B30] Stiles J , Jernigan TL. The basics of brain development. Neuropsychol Rev 2010;20:327–48. 21042938 10.1007/s11065-010-9148-4 PMC2989000

[vbae163-B31] Turner TN , Coe BP, Dickel DE et al Genomic patterns of de novo mutation in simplex autism. Cell 2017;171:710–22.e12. 28965761 10.1016/j.cell.2017.08.047 PMC5679715

[vbae163-B32] The UniProt Consortium. UniProt: the universal protein knowledgebase in 2021. Nucleic Acids Res 2021;49:D480–9. 33237286 10.1093/nar/gkaa1100 PMC7778908

[vbae163-B33] Vaswani A et al Attention is all you need. In: *Advances in Neural Information Processing Systems 30*, Long Beach, CA, USA. Red Hook, NY, United States: Neural Information Processing Systems (NIPS 2017), 2017.

[vbae163-B34] Whisstock JC , Lesk AM. Prediction of protein function from protein sequence and structure. Q Rev Biophys 2003;36:307–40. 15029827 10.1017/s0033583503003901

[vbae163-B35] Yang J , Yan R, Roy A et al The I-TASSER suite: protein structure and function prediction. Nat Methods 2015;12:7–8. 10.1038/nmeth.3213 PMC4428668 25549265

[vbae163-B36] You R , Huang X, Zhu S. Deep-Text2GO: improving large-scale protein function prediction with deep semantic text representation. Methods 2018;145:82–90. 29883746 10.1016/j.ymeth.2018.05.026

[vbae163-B37] You R , Yao S, Mamitsuka H et al DeepGraphGO: graph neural network for large-scale, multispecies protein function prediction. Bioinformatics 2021;37:i262–71. 34252926 10.1093/bioinformatics/btab270 PMC8294856

[vbae163-B38] Zhang J , Zhu M, Qian Y. protein2vec: predicting protein-protein interactions based on LSTM. IEEE/ACM Trans Comput Biol Bioinform 2022;19:1257–66. 32750870 10.1109/TCBB.2020.3003941

[vbae163-B39] Zhao F , Zhang C, Geng B. Deep multimodal data fusion. ACM Comput Surv 2024;56:1–36. 10.1145/3649447

[vbae163-B40] Zhou P et al Attention-based bidirectional long short-term memory networks for relation classification. In: *Proceedings of the 54th Annual Meeting of the Association for Computational Linguistics (volume 2: Short papers)*, Berlin, Germany. Stroudsburg, PA, USA: Association for Computational Linguistics, 2016, 207–12.

