# Correction to: RecGOBD: accurate recognition of gene ontology related brain development protein functions through multi-feature fusion and attention mechanisms

**DOI:** 10.1093/bioadv/vbaf065

**Published:** 2025-04-09

This is a correction to:

Zhiliang Xia, Shiqiang Ma, Jiawei Li, Yan Guo, Limin Jiang, Jijun Tang, RecGOBD: accurate recognition of gene ontology related brain development protein functions through multi-feature fusion and attention mechanisms, *Bioinformatics Advances*, Volume 4, Issue 1, 2024, vbae163, https://doi.org/10.1093/bioadv/vbae163

In the originally published version of this manuscript, there is an additional affiliation omitted for author, Zhiliang Xia.

The correction affiliation is:

1. Shenzhen Institute of Advanced Technology, Chinese Academy of Sciences , Shenzhen, Guangdong 518055, China

And

2. University of Chinese Academy of Sciences, Beijing 100049, China

This error has been corrected.

