# Supplementary material for: RecGOBD: accurate recognition of gene ontology related brain development protein functions through multi-feature fusion and attention mechanisms
Source: Bioinform Adv. 2024 Nov 4;4(1):vbae163. doi: 10.1093/bioadv/vbae163 (PMC11639192; doi:10.1093/bioadv/vbae163)
Supplement: vbae163_Supplementary_Data [file vbae163_supplementary_data.zip › SF1.pdf]

Supporting Information for

RecGOBD: Accurate Recognition of Gene Ontology Related Brain Development Protein Functions through Multi-Feature Fusion and Attention Mechanisms

Zhiliang Xia,[1] Shiqiang Ma,[1] Jiawei Li,[1] Yan Guo,[2] Limin Jiang[2],∗ and Jijun Tang[1],∗

1 Department, Shenzhen Institute of Advanced Technology, Chinese Academy of Sciences, Shenzhen, 518055, Guangdong, China and

2 Department of Public Health and Sciences, Sylvester Comprehensive Cancer Center, University of Miami, Miami, 33136, FL, USA

∗ Corresponding Email: jj.tang@siat.ac.cn, lxj423@med.miami.edu

1. Selected GO terms

We utilized a carefully curated dataset focused on proteins related to brain development. Initially, ontologies associated with brain development were selected from GO. Subsequently, protein sequences tagged with these ontologies were retrieved from the Uniprot database. These sequences were meticulously extracted and allocated into training, validation, and test sets in an 8:1:1 ratio. The selected GO terms are in Table 1.

Table 1.GO Terms and Categories.

GO ID	Categories	Terms
GO:0002250	Biological Process	adaptive immune response
GO:0004984	Molecular Function	olfactory receptor activity
GO:0006955	Biological Process	immune response
GO:0007420	Biological Process	brain development
GO:0042742	Biological Process	defense response to bacterium
GO:0045087	Biological Process	innate immune response
GO:0045471	Biological Process	response to ethanol
GO:0071277	Biological Process	cellular response to calcium ion
GO:0071456	Biological Process	cellular response to hypoxia
GO:0150104	Biological Process	transport across blood-brain barrier

2. Model Training

In our model, we use the PyTorch and TensorFlow frameworks. The pre-trained protein language models are implemented using the PyTorch 1.1.8 framework, while the rest of the models are built using TensorFlow 2.6. Additionally, we train our models using the Nvidia GeForce 3090. Our dataset consists of independent training, validation, and testing sets. We compute sigmoid outputs for ten labels related to brain development and use the Adam optimizer to minimize the loss function. Our model is fitted on the training set and hyperparameters are optimized on the validation set, with the final performance evaluated on an independent test set. The model employs early stopping, where training is halted if

the validation set loss does not decrease within five epochs, ensuring that the experiment does not overfit and retaining the model with the lowest loss for subsequent prediction and evaluation.

3. Evaluation Metrics

To comprehensively evaluate the performance of our model, we used three evaluation metrics: AUPR (Area Under the Precision-Recall Curve), AUROC (Area Under the Receiver Operating Characteristic Curve), and Fmax. AUPR is a crucial evaluation metric, particularly for handling imbalanced datasets, as it considers both precision and recall. Precision represents the proportion of true positives among the positive predictions, while recall measures the proportion of true positives predicted among all actual positive cases. On the other hand, AUROC, a widely used performance metric, assesses the model's diagnostic ability by plotting the relationship between the true positive rate (TPR) and the false positive rate (FPR). The area under the ROC curve (AUROC) provides an effective measure reflecting the overall performance of the model across different decision thresholds.Fmax is the maximum F-measure calculated for all prediction thresholds centered on proteins. First, we use the following formulas to calculate the average precision and recall:

$$pri(t) = \frac{\sum_f I(f \in P_i(t) \cap f \in T_i)}{\sum_f I(f \in P_i(t))}$$

$$rci(t) = \frac{\sum_f I(f \in P_i(t) \cap f \in T_i)}{\sum_f I(f \in T_i)}$$

$$\text{AvgPr}(t) = \frac{1}{m(t)} \sum_{i=1}^{m(t)} pri(t)$$

$$\text{AvgRc}(t) = \frac{1}{n} \sum_{i=1}^{n} rci(t)$$

where f is the GO term, T_i is a set of true annotations, $P_i(t)$ is a set of predicted annotations for protein i at threshold t, $m(t)$ is the number of proteins predicted to have at least one class, n is the total number of proteins, and I is an identity function that returns 1 if the condition is true, otherwise 0.

Next, we calculate the maximum F_{max} value for the prediction threshold:

$$F_{\text{max}} = \max_t \{\frac{2 \cdot \text{AvgPr}(t) \cdot \text{AvgRc}(t)}{\text{AvgPr}(t) + \text{AvgRc}(t)}\}$$

for thresholds $t \in [0,1]$ with a step size of 0.01. We identify the maximum F_{max} among these thresholds.

4. Optimizing Hyperparameters for Enhanced Model Performance.

In our experiments, we specifically focused on three key hyperparameters: learning rate, learning rate scheduler, and loss function, and we thoroughly explored their combined impact

on model performance (Table 2). We found that precisely adjusting the learning rate can significantly enhance the model's performance, especially when processing complex protein sequence data. Additionally, our experimental results revealed a crucial finding: a significant performance improvement can be achieved when four different embedding strategies are fused and applied to the model. Specifically, with the use of the Focal loss function along with a learning rate scheduler, the model achieved an AUROC of 0.917,an AUPR of 0.694 and an Fmax of 0.689. On the other hand, when employing the Binary Cross-Entropy (BCE) loss function, the AUROC slightly decreased to 0.914, but the AUPR increased to 0.697 and the Fmax increased to 0.739. These results suggest that after fusing multiple embedding strategies, using a higher learning rate combined with a BCE loss function can yield better outcomes for the model.

Table 2.The performance of different embeddings under two types of loss functions.

Model	Loss	Scheduler	Learning Rate	AUROC	AUPR	Fmax
onehot	BCE	None	0.001	0.782	0.427	0.494
onehot	Focal	None	0.001	0.616	0.265	0.409
protein2vec	BCE	None	0.001	0.834	0.51	0.481
protein2vec	Focal	None	0.001	0.788	0.452	0.473
ProtBert	BCE	None	0.001	0.902	0.614	0.627
ProtBert	Focal	None	0.001	0.905	0.628	0.564
ESM2	BCE	None	0.001	0.909	0.683	0.702
ESM2	Focal	None	0.001	0.908	0.665	0.699
ESM2	BCE	None	0.0005	0.905	0.66	0.705
ESM2	BCE	StepLR	0.001	0.909	0.677	0.715
ESM2+onehot	BCE	None	0.001	0.909	0.67	0.697
ESM2+onehot	Focal	None	0.001	0.912	0.683	0.701
ESM2+ProtBert	BCE	None	0.001	0.913	0.68	0.729
ESM2+ProtBert	Focal	None	0.001	0.912	0.681	0.726
ESM2+ProtBert	BCE	None	0.0005	0.911	0.688	0.635
ESM2+ProtBert	BCE	StepLR	0.001	0.915	0.693	0.715
ESM2+Protein2vec+onehot	BCE	None	0.001	0.913	0.684	0.701
ESM2+Protein2vec+onehot	Focal	None	0.001	0.909	0.673	0.627
ESM2+ProtBert+onehot	BCE	None	0.001	0.911	0.667	0.717
ESM2+ProtBert+onehot	Focal	None	0.001	0.912	0.667	0.712
ESM2+ProtBert+onehot	Focal	None	0.0005	0.911	0.685	0.66
ESM2+ProtBert+onehot	Focal	StepLR	0.001	0.913	0.675	0.678
ESM2+ProtBert+protein2vec+onehot	BCE	None	0.001	0.914	0.697	0.739
ESM2+ProtBert+protein2vec+onehot	Focal	None	0.001	0.915	0.686	0.666
ESM2+ProtBert+protein2vec+onehot	Focal	None	0.0005	0.913	0.683	0.643
ESM2+ProtBert+protein2vec+onehot	Focal	StepLR	0.001	0.917	0.694	0.689

5. Comparative Analysis of Correlation for Embedding Technologies.

To delve into the differences among various pre-trained models (onehot, Protein2vec, ProtBert, and ESM2), we analyzed the protein sequence embeddings generated by these models. Initially, we set the maximum length of the protein sequences to 2000 and applied appropriate padding or truncation as needed. Then, we flattened the obtained embeddings into a two-dimensional array (with dimensions equal to the number of sequences multiplied by 2000 times the embedding length) and calculated the cosine similarity matrix of the embeddings output by each model. Using these data, we generated heatmaps and dendrograms for each pre-trained model FigureS1.

In the heatmaps, the horizontal and vertical axes represent the indices of protein sequences, and the colors represent the cosine similarity between the corresponding proteins. The colors range from light green to dark blue, where lighter colors indicate lower similarity and darker colors indicate higher similarity. The dendrograms above and to the left of the heatmaps show the hierarchical clustering relationships between proteins. The length of the branches represents the clustering distance, with shorter branches indicating higher similarity and longer branches indicating lower similarity.

From these visualizations, we can clearly see the similarity of the embeddings generated by different models. For example, the embeddings generated by the ESM model show higher similarity, indicating that the ESM model captures more connections between sequences, thus achieving superior predictive performance. Specifically, the heatmap of the ESM model shows more dark blue regions, indicating higher similarity between the protein embeddings it generates. The shorter branches in the dendrogram further support this, showing the ESM model's advantage in capturing the similarity between protein sequences.

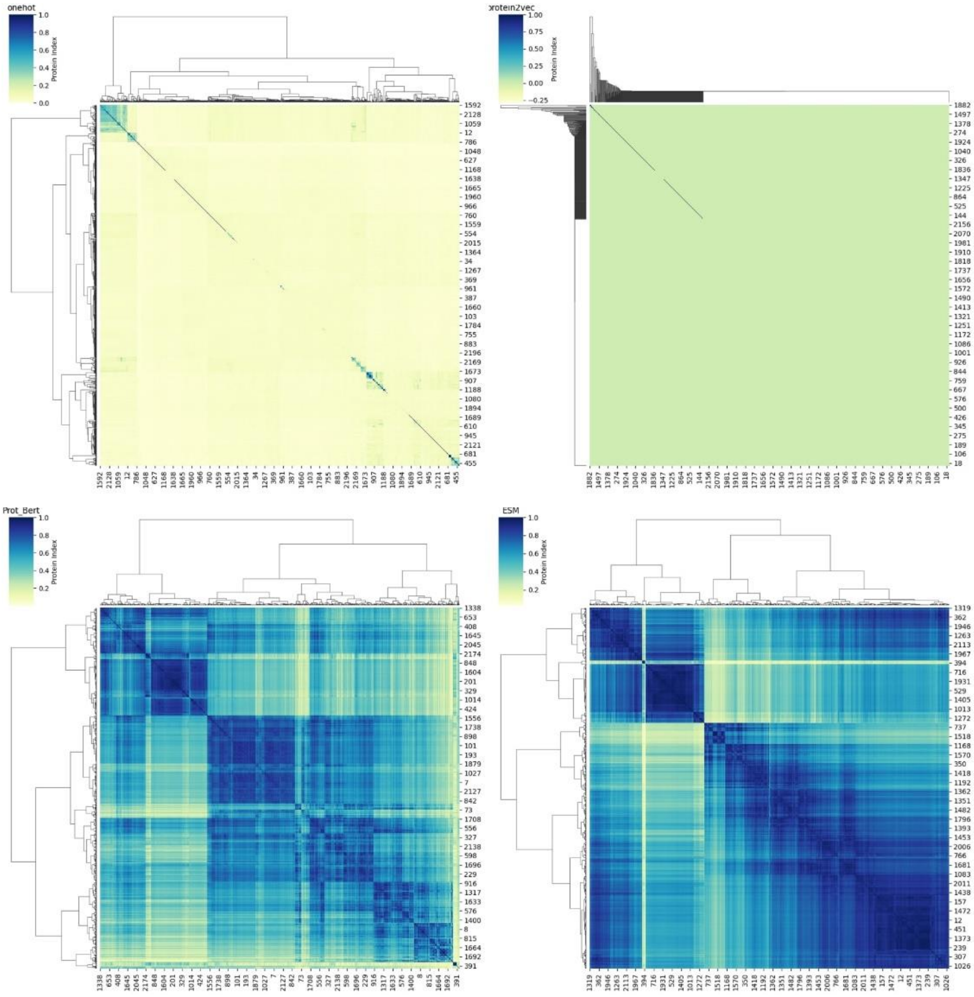

FigureS1.The four heatmaps above represent the association heatmaps and clustering information of protein sequences encoded by four different embeddings (onehot, protein2vec, ProtBert and ESM). In the heatmaps, the horizontal and vertical axes represent the indices of protein sequences, and the colors represent the cosine similarity between the corresponding proteins. The colors range from light green to dark blue, with lighter colors indicating lower similarity and darker colors indicating higher similarity. The dendrograms above and to the left of the heatmaps show the hierarchical clustering relationships between proteins. The length of the branches represents the clustering distance, with shorter branches indicating higher similarity and longer branches indicating lower similarity.

6. Specific GO performance analysis

In this section, we perform a performance validation of functional predictions for protein sequences using well-trained models. With protein sequences as input, these models generate prediction probabilities for each protein function GO label. To visually evaluate and compare the predictive performance of different models, we adopted the method of plotting ROC curves. ROC curves are an important tool for assessing classifier performance, providing a visual means to evaluate model predictions by plotting the False Positive Rate (FPR) against the True Positive Rate (TPR). In the context of ROC curves, the TPR, also known as sensitivity, indicates the model's ability to correctly identify positive cases; the FPR represents the

proportion of negative cases incorrectly identified as positive. An ideal prediction model aims to be as close to the top left corner of the plot as possible, achieving high TPR while maintaining a low FPR, resulting in an ROC curve that bends towards the upper left corner. The AUC serves as a metric to quantify model performance. AUC values range from 0 to 1, where higher values (closer to 1) indicate better model performance, effectively distinguishing between positive and negative classes. For each GO label, we showcased the ROC curves for protein function predictions made by different pre-trained models and their combined models (Figure S2). This not only allows us to compare the performance differences between individual models but also enables us to assess the impact of model fusion on prediction outcomes. Through this approach, we can identify which models are more suitable for addressing specific protein function prediction challenges.

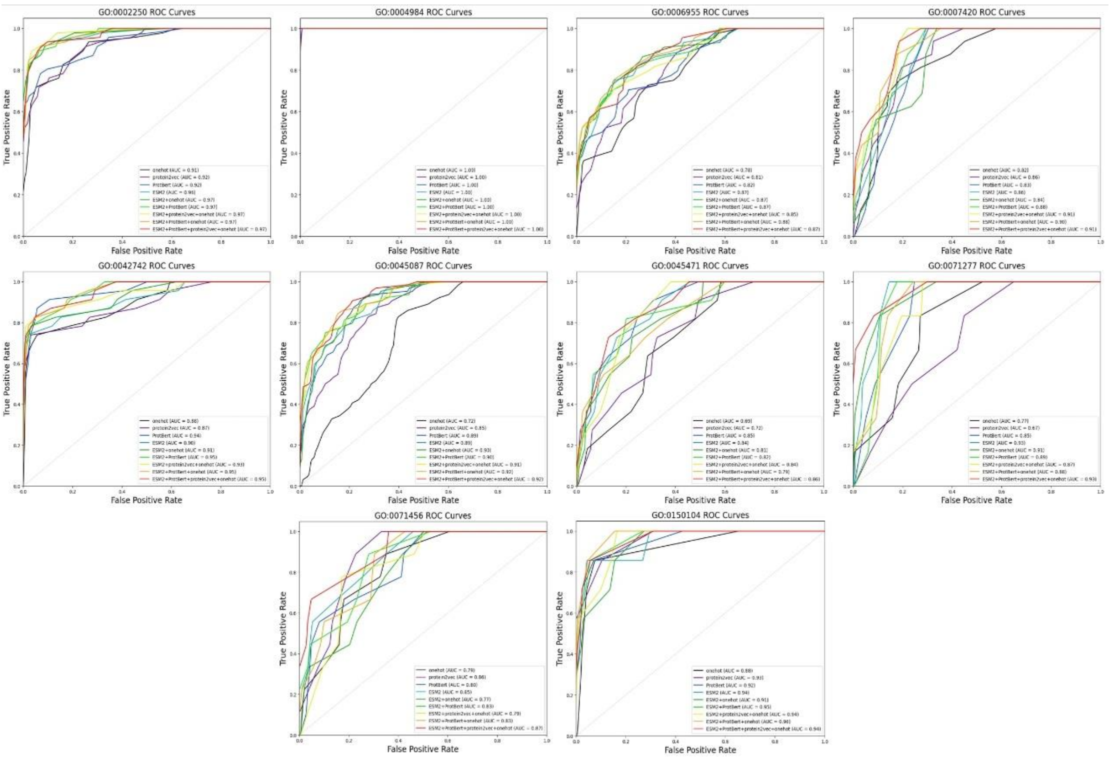

FigureS2. Specific GO performance results for RecGOBD. For each GO label, we separately showcased the effects of functional predictions for protein sequences encoded by different pre-trained models through a deep learning framework. Notably, by integrating four distinct pre-trained models, we observed that the area under the curve (AUC) values obtained were generally higher, significantly outperforming the performance of individual models. This outcome distinctly illustrates that a multi-model fusion strategy can substantially enhance prediction accuracy in protein function prediction tasks, as depicted in the figure.
